# DALI: Defining Antibiotic Levels in Intensive care unit patients: a multi-centre point of prevalence study to determine whether contemporary antibiotic dosing for critically ill patients is therapeutic

**DOI:** 10.1186/1471-2334-12-152

**Published:** 2012-07-06

**Authors:** Jason A Roberts, Jan J De Waele, George Dimopoulos, Despoina Koulenti, Claude Martin, Philippe Montravers, Jordi Rello, Andrew Rhodes, Therese Starr, Steven C Wallis, Jeffrey Lipman

**Affiliations:** 1 Burns Trauma and Critical Care Research Centre, The University of Queensland, Brisbane, Australia; 2 Royal Brisbane and Women’s Hospital, Brisbane, Australia; 3 Ghent University Hospital, Ghent, Belgium; 4 Attikon University Hospital, Athens, Greece; 5 Hospital Nord, Marseille, France; 6 Centre Hospitalier Universitaire Bichat-Claude Bernard, AP-HP, Université Paris VII, Paris, France; 7 Hospital Vall d’Hebron, Barcelona, Spain; 8 St George’s Healthcare NHS Trust and St George’s University of London, London, England, UK

**Keywords:** Antibiotic, βeta-lactam, Glycopeptide, Triazole, Echinocandin, Continuous infusion, Extended infusion, Bolus dosing, Adverse events, Pharmacokinetics, Pharmacodynamics

## Abstract

**Background:**

The clinical effects of varying pharmacokinetic exposures of antibiotics (antibacterials and antifungals) on outcome in infected critically ill patients are poorly described. A large-scale multi-centre study (DALI Study) is currently underway describing the clinical outcomes of patients achieving pre-defined antibiotic exposures. This report describes the protocol.

**Methods:**

DALI will recruit over 500 patients administered a wide range of either beta-lactam or glycopeptide antibiotics or triazole or echinocandin antifungals in a pharmacokinetic point-prevalence study. It is anticipated that over 60 European intensive care units (ICUs) will participate. The primary aim will be to determine whether contemporary antibiotic dosing for critically ill patients achieves plasma concentrations associated with maximal activity. Secondary aims will compare antibiotic pharmacokinetic exposures with patient outcome and will describe the population pharmacokinetics of the antibiotics included. Various subgroup analyses will be conducted to determine patient groups that may be at risk of very low or very high concentrations of antibiotics.

**Discussion:**

The DALI study should inform clinicians of the potential clinical advantages of achieving certain antibiotic pharmacokinetic exposures in infected critically ill patients.

## Background

Effective antibiotic treatment of intensive care unit (ICU) patients that have overwhelming infections, including sepsis, severe sepsis and septic shock, remains a significant challenge to physicians world-wide [1-7]. Therapy that is not initiated rapidly or with sufficient antibiotic spectrum increases in-hospital mortality [3,4]. Indeed, sepsis itself has an incidence in the population that exceeds colon cancer, breast cancer, and AIDS, with mortality rates of 30% for mild to moderate sepsis and up to 82% for severe sepsis and septic shock [5]. Despite advances in critical care medicine, the incidence of sepsis continues to increase and the prognosis remains poor. Although there has been significant investment into treatments that limit the various inflammatory and coagulation cascades, none of these therapies have been able to demonstrate the same outcome benefits as effective antibiotic therapy [3,8]. It is thought that optimisation of antibiotic dosing may well further improve clinical outcomes for ICU patients with infections.

Drug doses are usually derived from healthy volunteers and then extrapolated into ICU patients. A challenge for clinicians is that standard dosing in ICU patients does not achieve the same concentrations seen in non-critically ill patients [9]. There is a significant body of literature that demonstrates that disease-processes experienced by critically ill patients frequently cause pharmacokinetic changes that may result in either sub-therapeutic or toxic drug concentrations of antibiotics (including antibacterial and antifungal drugs) [9-17]. Given the increased level of resistance of bacteria in the ICU [18], and these potentially lower antibiotic exposures, treatment failure of infections is unsurprisingly common. Subtherapeutic drug concentrations may also promote selection of resistant microorganisms, further adding to the threat of antibiotic resistance in the ICU. To address this issue innovative approaches to dosing may be required to ensure optimal drug exposures [19-21].

Although altered antibiotic concentrations have been accurately described in various critically ill patient sub-populations in small research studies [21-28], there is no large multi-centre evaluation that seeks to determine whether the issues identified in a controlled research environment correspond to clinical practice. Such an evaluation is essential for determining whether action is required to change existing global antibiotic prescribing practices for critically ill patients. If prescribing should be found to be sub-optimal, then the motivation for changes to existing prescribing practice may lead to improved clinical cure rates and a reduction in the rate of antibiotic resistance in the critical care environment. To address the insufficiency of data available to clinicians on the adequacy of empiric antibiotic dosing in ICU patients, on behalf of the Infection Section of the European Society of Intensive Care Medicine, the authors proposed a multicentre point-prevalence pharmacokinetic study in ICU patients.

This proposal has been supported by the European Society of Intensive Care Medicine’s European Critical Care Research Network (ESICM ECCRN) and Trials Group and also the the Royal Brisbane and Women’s Hospital Research Foundation (Australia). This report describes the study protocol.

Based on our previous research in many different critically ill patient sub-populations, we hypothesize that 40 to 70% of critically ill patients are receiving suboptimal antibiotic dosing [23,27-33].

### Methods/Design

The Defining Antibiotic Levels in Intensive care unit patients (DALI) study is a prospective, multi-centre pharmacokinetic point-prevalence study describing whether contemporary antibiotic dosing in ICU patients achieves concentrations associated with maximal activity. It is anticipated that the study will recruit over 500 ICU patients from over 60 ICUs throughout 10 countries in Europe over a one-week period. The primary and secondary aims as well as the proposed sub-group analyses are as follows:

Primary aim

To determine whether contemporary antibiotic dosing for critically ill patients achieves concentrations associated with maximal activity.

Secondary aims

Comparison of observed antibiotic pharmacokinetics/pharmacodynamics with the clinical outcome of therapy

Description of the population pharmacokinetics of the individual antibiotics in ICU patients

The proposed subgroups for the primary and secondary aims are:

Patients administered intermittent dosing versus extended or continuous infusions and

Patients with ‘steady-state’ versus ‘non-steady-state’ pharmacokinetics (‘non-steady-state’ defined as antibiotics commenced within 24-h prior to sampling)

Patients with different levels of sickness severity as measured by Sequential Organ Failure Assessment (SOFA) Score [34], Acute Physiology and Chronic Health Evaluation (APACHE) II Score [35] and PIRO (Predisposition, Infection, Response, Organ dysfunction) Score [36]

Different admission diagnoses

Different indications for antibiotic therapy

Presence of surgery within the 24-hours prior to sampling

Different total body weight

Different levels of renal function and presence of extracorporeal renal support techniques

### Participants

Identification of eligible patients will occur on a designated day (preferably Monday) of a nominated week. Informed consent is required from each patient or a legally authorised representative to participate in the study. Participants would need to fulfil all the inclusion and exclusion criteria to be enrolled:

Inclusion criteria

Written informed consent has been obtained from the patient or their legally authorised representative

Age ≥ 18 years

Receiving antibiotic therapy of one of the target drugs via continuous or intermittent dosing regimen

Suitable intravenous/intra-arterial access to facilitate sample collection

Exclusion criteria

Consent not obtained

Aged < 18 years of age

Not being administered any of the study antibiotics

Limited or no intravenous/intra-arterial access.

### Study treatments and pharmacokinetic sampling

With the exception of blood sampling, there is no intervention in this study that may affect patient treatment. Antibiotic dosing will occur as deemed by the treating clinician and their local dosing practices. Patients receiving the study antibiotics will be identified on the nominated week for pharmacokinetic sampling. During a single dosing interval of that week, each patient will then have two blood samples taken for each antibacterial agent and/or three blood samples for antifungals (Table 1). For patients on multiple study drugs, each drug will be sampled independent of the other drug(s). Table 1 outlines the test antibiotics (antibacterials and antifungals) to be sampled, the timing of pharmacokinetic sampling and the pharmacodynamics endpoints that will be tested for each antibiotic and dosing regimen.

**Table 1:** Study drugs, routes of administration, pharmacokinetic/pharmacodynamic targets and blood sampling

**Study Drugs and method of infusion**	**Pharmacokinetic Sampling**	**Pharmacodynamic Targets tested**
**Beta-lactam antibiotics** by intermittent infusion	Sample A: mid-way through dosing interval (50% of dosing interval)	· 50% *f* T_>MIC_
(amoxycillin-clavulanate; ampicillin; piperacillin-tazobactam; penicillin-G; flucloxacillin; dicloxacillin; cloxacillin; cephazolin; ceftazidime; ceftriaxone; cefepime; meropenem; imipenem; doripenem; ertapenem)	Sample B: within 30 min of next dose (100% of dosing interval)	· 50% *f* T_>4xMIC_*
**Glycopeptide antibiotics** by intermittent infusion (vancomycin, teicoplanin)		· 100% *f* T_>MIC_
		· 100% *f* T_>4xMIC_
		· 100% *f* T_>4xMIC_
		· Concentration ≥15 mg/L*
**Triazole antifungals** (fluconazole, voriconazole)	Sample A: 30 min after completion of intravenous infusion (peak concentration)	· AUC_0-24_/MIC ≥25*
**Echinocandin antifungals** (caspofungin, micafungin, anidulafungin)	Sample B: mid-way through dosing interval (50% of dosing interval)	· AUC_0-24_/MIC ≥20*
	Sample C: within 30 min of next dose (100% of dosing interval)	
**Beta-lactam antibiotics** (listed above) by continuous infusion	Sample A: at any time	· 100% *f* T_>4xMIC_*
	Sample B: >6 hours after sample A	
**Glycopeptide antibiotics** (listed above) by continuous infusion		· 100% *f* T_>4xMIC_*
		· AUC_0-24_/MIC ≥350

* denotes the primary endpoint – other stated pharmacodynamics targets are secondary endpoints.

*f* T_>MIC_ is the duration of a dosing interval for which the antibiotic concentration remains above the minimum inhibitory concentration (MIC) of the known or suspected pathogen (endpoints of 50% or 100% of the interval, and MIC is defined by EUCAST MIC_90_ data); *f* T_>4xMIC_ is the duration of a dosing interval for which the antibiotic concentration remains above a concentration that is 4 x the MIC of the known or suspected pathogen (endpoints of 50% or 100% of the interval, and MIC is defined by EUCAST MIC_90_ data); AUC_0-24_/MIC is the ratio of the area under the concentration time curve from 0–24 hours to MIC.

The blood sampling has been designed to determine drug concentrations at various time points to describe whether pharmacokinetic/pharmacodynamic targets are achieved in individual patients (Table 1). To achieve the endpoint 50% *f* T_>MIC_, sample A is taken mid-way through the dosing interval to see if the drug concentration exceeds the MIC. The 100% *f* T_>MIC_ endpoint is similarly assessed from the sample B taken at the end of the dosing interval. The endpoint 100% *f* T_>4xMIC_, is attained if all sample concentrations exceed the MIC by at least a factor of four. For the parameter AUC_0-24_/MIC, the Area Under the concentration-time Curve from 0–24 h (AUC_0-24_) is calculated by the trapezoidal rule, and to attain the stated endpoint, the AUC_0-24_ must exceed the MIC by the factor listed in Table 1. Where the MIC not known, the MIC of the infecting pathogen will be defined by The European Committee on Antimicrobial Susceptibility Testing (EUCAST) MIC_90_ data; available at: http://www.eucast.org/clinical_breakpoints).

### Data collection and management

Data collection will be conducted by trained staff at each participating centre and entered onto a case report form (CRF). At the end of the patient’s participation, the CRF will be sent to the coordinating centre (The University of Queensland, Australia). Outstanding queries regarding the completion of the CRF will be undertaken with each participating centre where necessary to ensure accuracy of data.

The data to be collected includes.

Demographic data

Age

Gender

Height

Weight

Clinical data

Admission diagnosis,

Sickness severity scores (APACHE II, SOFA, PIRO)

Presence of extracorporeal circuits (e.g. RRT (renal replacement therapy), ECMO (extracorporeal membrane oxygenation))

Procalcitonin (where available),

Presence/absence of surgery within previous 24 hours

Clinical outcome of infection

Mortality at 30-days

Organ function data

Renal function – serum creatinine concentration during studied dosing interval; MDRD (modified diet in renal disease) equation

8-hours urinary creatinine clearance (where available)

Fluid balance for total length of stay and previous 24-hours

Antibiotic dosing data

Dose and frequency

Time of dosing and sampling

Day of antibiotic therapy

Infection data

Known or presumed pathogen

Known or likely minimum inhibitory concentration (MIC)

The definitions used to assess clinical outcome of therapy are as follows. A positive clinical outcome of therapy is defined as completion of treatment course without change or addition of antibiotic therapy, and with no additional antibiotics commenced with 48 h of discontinuation of the antibiotic therapy.

De-escalation is defined as the change to a narrower spectrum antibiotic based on patient-specific microbiological data in the absence of clinical failure. For antibiotics that are ceased before the end of treatment of an infection because of antibiotic de-escalation, these antibiotics will be excluded from the *a priori* analysis. Where de-escalation occurs during the target week of sampling, an option for a second sampling period of the new antibiotic will exist to confirm appropriateness of antibiotic concentrations and compare these with the patient’s clinical outcome.

Safety data will be collected to define any adverse drug reaction (clinically observed, haematological or biochemical) that is reported by the clinical staff at the participating ICUs that is suspected as being caused by any of the study antibiotics.

### Maintenance of blood sample integrity

Blood samples will be kept on ice, centrifuged at 3000 rpm for 10-min, within 6 h of collection and the plasma transferred to a labelled cryo-vial for frozen storage (at −20°C or lower for short term storage). A commercial courier company specialising in transport of clinical samples on dry ice will collect the samples from each site and deliver to the Burns Trauma and Critical Care Research Centre at The University of Queensland, Australia for bioanalysis. Samples will be stored at −80°C until assay.

### Bioanalysis

The concentration of the study antibiotics in the biological samples will be determined by chromatographic methods (HPLC and LC-MS/MS) that are validated and conducted in accordance with the US Food and Drug Administration’s guidance for industry on bioanalysis (available at: http://www.fda.gov/downloads/Drugs/GuidanceComplianceRegulatoryInformation/Guidances/UCM070107.pdf).

### Ethical issues

Each of the participating centres has obtained local ethics approvals to conduct the study as described in Additional file 1. The University of Queensland is the head institution with ethical approval granted by the Medical Research Ethics Committee (201100283 12th April 2011 and Amendment 201100283 25th May 2011). Patients may withdraw from the study at any time without prejudice, as documented and explained at the time of consenting.

### Statistical and pharmacokinetic analysis

The achievement of the pharmacodynamics targets will be performed by visual inspection of the results and comparison with the target. Statistical analyses to test the study objectives will be performed using Mann–Whitney U tests or Students t-tests where appropriate using the statistical package, SPSS (version 17.0, Illinois, USA).

The % *f* T_>MIC_ will be determined using the equation [37]:

$$\%fT_{>\text{MIC}}=\text{ln}\left[\text{Dose}/\left(V_{\text{d}}\times \text{MIC}\right)\right]\times \left(1/k_{\mathrm{el}}\right)\times \left(100/\text{DI}\right)$$

where V_d_ is volume of distribution calculated as Dose/AUC; MIC is the known or suspected minimum inhibitory concentration; kel is the elimination rate constant calculated from the gradient of the concentration-time curve in the elimination phase (sample A and sample B) and DI is the dosing interval (h). Where infections are polymicrobial, the MIC of the least susceptible pathogen will be used in the analysis.

The population pharmacokinetic parameters of each antibiotic will be determined using a population pharmacokinetic modelling approach using NONMEM® (Version 6.1, GloboMax LLC, Hanover, MD, USA) as previously described [22,23,25,29,32,38]. Additionally, the pharmacokinetic model will aim to determine if significant correlations exist between demographic and clinical factors on pharmacokinetics. If one or more of the variables are found to have a significant effect on the pharmacokinetics of the drug, then it can be incorporated into the final pharmacokinetic model.

### Sample size and power

Whilst it is not possible to predict the number of patients receiving each of the study antibiotics, all data will be useful and can be used to inform clinical practice. It is likely that at least 5 of the study antibiotics will have a minimum of 30 patients included in the analysis. This sample size will provide a power of 80% (assuming an α of 0.05 and r^2^ of 30%) for defining at least 2–4 covariates predictive of achieving the primary pharmacodynamic outcome [39]. For all other included study antibiotics, we estimate that each will have a minimum of 12 patients that can be used for the secondary objectives of population pharmacokinetic analysis. A minimum of 12 patients per antibiotic is based on data from previous non-interventional pharmacokinetic studies in critically ill patients [22,23,32,40].

### Funding

This project has received funding from the European Society of Intensive Care Medicine’s European Critical Care Research Network (ESICM ECCRN), and the Royal Brisbane and Women’s Hospital Foundation. Dr Roberts is funded by a Training Research Fellowship from the National Health and Medical Research Council of Australia (569917).

## Discussion

ICU patients are greatly different in many ways to non-critically ill patients. Principal differences relate to the level of sickness severity, the number of therapeutic interventions used, the severe pathophysiological changes that occur and the presence of highly resistant bacteria and fungi. For these reasons, it unsurprising that ICU patients have poor outcomes associated with infections. Whilst early and appropriate treatment of infections significantly reduces patient mortality, the additional benefits of optimised antibiotic pharmacokinetic exposures have been poorly quantified. ICU pharmacokinetic studies have traditionally only enrolled small patient numbers, which greatly limits the ability to describe the significant interpatient pharmacokinetic variability that is present, and what effect this may have on clinical efficacy. Using a multinational approach to enrol large patients numbers on a wide range of commonly used antibiotics, including both antibacterials and antifungals, the DALI study will address these knowledge gaps.

## Abbreviations

AIDS: Acquired Immunodeficiency syndrome; APACHE: Acute physiology and chronic health evaluation; AUC: Area under the concentration-time curve; AUC_0-24_/MIC: Area under the concentration-time curve from 0–24 h; CRF: Case report form; DALI: Defining Antibiotic Levels in Intensive care patients; DI: Dosing interval; ECCRN: European Critical Care Research Network; ECMO: Extracorporeal membrane oxygenation; ESICM: European Society of Intensive Care Medicine’; *f* T_>MIC_: The duration of a dosing interval for which the antibiotic concentration remains above the MIC of the known or suspected pathogen; *f* T_>4xMIC_: The duration of a dosing interval for which the antibiotic concentration remains above a concentration that is 4 × the MIC of the known or suspected pathogen; HPLC: High performance liquid chromatography; ICU: Intensive care unit; kel: Elimination rate constant; LC-MS/MS: Liquid chromatography tandem mass spectrometry; MDRD: Modified diet in renal disease; MIC: Minimum inhibitory concentration; MIC_90_: Concentration required to inhibit the growth of 90% of organisms after 24-h; PIRO: Predisposition, infection, response, organ dysfunction; RRT: Renal replacement therapy; SOFA: Sequential organ failure assessment; V_d_: Volume of distribution.

## Competing interests

The authors declare that they have no competing interests.

## Authors’ contribution

JAR and JL designed the study and wrote the initial protocol. JJD, GD, DK, CM, PM, JR, AR TS and SCW TS provided advice and input into the protocol. All authors read and approved the final manuscript. This study was partly funded by the European Society of Intensive Care Medicine from which it received a grant (see below).

## Pre-publication history

The pre-publication history for this paper can be accessed here:

http://www.biomedcentral.com/1471-2334/12/152/prepub

## Supplementary Material

Additional file 1 The location of the participating sites (country and city) and Ethics committee approving conduct of the study in each site. Click here for file
