# Supplementary material for: DALI: Defining Antibiotic Levels in Intensive care unit patients: a multi-centre point of prevalence study to determine whether contemporary antibiotic dosing for critically ill patients is therapeutic
Source: BMC Infect Dis. 2012 Jul 6;12:152. doi: 10.1186/1471-2334-12-152 (PMC3506523; doi:10.1186/1471-2334-12-152)
Supplement: Additional file 1 — The location of the participating sites (country and city) and Ethics committee approving conduct of the study in each site. [file 1471-2334-12-152-S1.docx]

| **Country** | **City** | **Hospital** | **Ethics Committee** |
| --- | --- | --- | --- |
| **Andorra** | Escaldes-Engordany | 1. Hospital Nostra Senyora de Meritxell | Hospital Nostra Senyora de Meritxell |
|  | | | |
| **Belgium** | Antwerp | 2. Universitair Ziekenhuis Antwerpen | Commissie Voor Medische Ethiek, Universitair Ziekenhuis Gent |
|  | Antwerp | 1. Algemeen Ziekenhuis Monica |  |
|  | Brussels | 1. St Luc Brussels |  |
|  | Brussels | 1. Universitair Ziekenhuis Brussels |  |
|  | Brussels | 1. Erasme University Hospital |  |
|  | Ghent | 1. Ghent University Hospital |  |
|  | Leuven | 1. Universitair Ziekenhuis Gasthuisberg |  |
|  | Ottignies | 1. St Pierre |  |
|  | Ottignies | 1. Clinique Saint Pierre |  |
|  | | | |
| **Finland** | Helsinki | 1. Helsinki University Hospital | Helsinki University Hospital |
|  | | | |
| **France** | Angers | 1. Centre Hospitalo Universitaire d’Angers | Agence francaise de securite des produits de sante |
|  | Aix en Provence | 1. Centre Hospitalier du Pays d’Aix |  |
|  | Amiens | 1. Centre Hospitalo Universitaire d’Amiens |  |
|  | Clermont Ferrand | 1. Centre Hospitalo Universitaire d’Estaing |  |
|  | Grenoble | 1. Hôpital Michalon |  |
|  | Marseille | 1. Hospital Nord – Pavillon Etoile |  |
|  | Montpellier | 1. Hôpital St Eloi |  |
|  | Nice | 1. Hôpital St Roch |  |
|  | Nimes | 1. Centre Hospitalo Universitaire de Nimes Caremeau |  |
|  | Paris | 1. Centre Hospitalo Universitaire Bichat - Claude Bernard |  |
|  | Pessac | 1. Centre Hospitalo Universitaire de Bordeaux |  |
|  | Rennes | 1. Hopital Pontchaillou |  |
|  | Strasbourg | 1. Nouvel Hopital Civil |  |
|  | | | |
| **Great Britain** | London | 1. Imperial College: Charing Cross | National Research Ethics Service (NRES) London - Harrow |
|  | London | 1. Imperial College: Hammersmith |  |
|  | London | 1. Imperial College: St Marys Hospital |  |
|  | London | 1. St George's Hospital Medical School |  |
|  | | | |
| **Greece** | Athens | 1. Attikon University Hospital | Attikon University Hospital |
|  | Alexandroupolis | 1. University Hospital of Alexandroupolis Democritus | University Hospital of Alexandroupolis Democritus |
|  | Athens | 1. 251 Air Force General Hospital | 251 Air Force General Hospital |
|  | Athens | 1. General Hospital of Athens ‘Hippokrateion’ | General Hospital of Athens ‘Hippokrateion’ |
|  | Athens | 1. ‘Aghioi Anargyroi’ Hospital | ‘Aghioi Anargyroi’ Hospital |
|  | Athens | 1. ‘Sotiria’ General Hospital | ‘Sotiria’ General Hospital |
|  | Athens | 1. ‘Thriassio’ General Hospital of Eleusis | ‘Thriassio’ General Hospital of Eleusis |
|  | Athens | 1. ‘Aretaieion’ University Hospital | ‘Aretaieion’ University Hospital |
|  | Heraklion | 1. University Hospital Heraklion | University Hospital of Heraklion |
|  | Ioannina | 1. University Hospital of Ioannina | University Hospital of Ioannina |
|  | Larisa | 1. General Hospital of Larisa | General Hospital of Larisa |
|  | Thessaloniki | 1. General Hospital of Thessaloniki ‘G. Papageorgiou’ | General Hospital of Thessaloniki ‘G. Papageorgiou’ |
|  | Thessaloniki | 1. General Hospital of Thessaloniki ‘Hippokrateion’ | General Hospital of Thessaloniki ‘Hippokrateion’ |
|  | Thessaloniki | 1. ‘Ahepa’ University Hospital | ‘Ahepa’ University Hospital |
|  | Thessaloniki | 1. ‘G. GENNIMATAS’ General Hospital of Thessaloniki | ‘G. GENNIMATAS’ General Hospital of Thessaloniki |
|  | Trikala | 1. General Hospital of Trikala | General Hospital of Trikala |
|  | Athens | 1. Naval Hospital of Athens | Naval Hospital of Athens |
|  | Athens | 1. ‘Aghia Olga-Konstantopouleion’ General Hospital | ‘Aghia Olga-Konstantopouleion’ General Hospital |
|  | Athens | 1. General Hospital of Athens ‘NIMITS’ | General Hospital of Athens ‘NIMITS’ |
|  | | | |
| **Italy** | Bologna | 1. Azienda Ospedaliera Universitaria Palermo | Santa maria della Misericordia |
|  | Firenze | 1. Azienda ospedaliero-universitaria Policlinico Sant Orsola Malpighi |  |
|  | Genoa | 1. Azienda Ospedaliero Universitaria Careggi |  |
|  | Palermo | 1. Ospedale San Martino Genova |  |
|  | Turin | 1. San Giovanni-Battista Molinette |  |
|  | | | |
| **Portugal** | Porto | 1. Hospital de Santo António | Centro Hospitalar de lisboa central, EPE Appreciacao do estudo clinico |
|  | | | |
| **Spain*** | Barcelona | 1. Hospital Vall d'Hebron | Comunidad Autónoma de Catalunya |
|  | Barcelona | 1. Hospital Del Mar |  |
|  | Barcelona | 1. Centro Médico Delfos |  |
|  | Barcelona | 1. Hospital General de L’Hospitalet |  |
|  | Barcelona | 1. Hospital General de Granollers |  |
|  | Barcelona | 1. Hospital Clinic Barcelona |  |
|  | Burgos | 1. Hospital General Yagüe | Comunidad Autónoma de Castilla y León |
|  | Madrid | 1. Hospital Universitario 12 de Octubre | Comunidad Autónoma de Madrid |
|  | Madrid | 1. Hospital Ramon y Cajal |  |
|  | Madrid | 1. Hospital Universitario La Paz |  |
|  | Madrid | 1. University Hospital Severo Ochoa |  |
|  | San Sebastián | 1. Hospital Donostia | Comunidad Autónoma del País Vasco |
|  | Santander | 1. Hospital Universitario Marques de Valdecilla | Comunidad Autónoma de Cantabria |
|  | Vitoria | 1. Hospital  Txagorritxu | Comunidad Autónoma del País Vasco |
|  | | | |
| **Turkey** | Ankara | 1. Hacettepe University | Hacettepe University |

* All Spanish sites had a central Spanish Ethics Approval from *Agencia Española del Medicamento y Producto Sanitario* as well as from the Ethics Committee in the Spanish Region.
